# Hidden in Plain Sight: Esophageal Dysmotility in Patients With Systemic Lupus Erythematosus

**DOI:** 10.7759/cureus.23208

**Published:** 2022-03-16

**Authors:** Alla Turshudzhyan, Abu Fahad Abbasi, Promila Banerjee

**Affiliations:** 1 Internal Medicine, University of Connecticut Health, Farmington, USA; 2 Internal Medicine, Loyola University Medical Center, Maywood, USA; 3 Gastroenterology and Hepatology, Hines VA Hospital, Hines, USA; 4 Gastroenterology and Hepatology, Loyola University Chicago Stritch School of Medicine, Maywood, USA

**Keywords:** egjoo, iem, egd, endoscopy, hrm, absent contractility, sle, esophageal dysmotility

## Abstract

Among the patients who present to the emergency room or a primary care office with symptoms of dysphagia, chest pain, and reflux, approximately 9% have an underlying rheumatological condition. It is not surprising that many emergency and internal medicine clinicians frequently overlook this etiology and investigate other causes first. However, an overwhelming number of patients with rheumatological conditions (61.1%) have some form of esophageal dysmotility that ranges from ineffective esophageal motility (IEM) to achalasia. We present a case of systemic lupus erythematosus (SLE) with absent contractility that was initially overlooked. Missing and/or absent contractility or other forms of esophageal dysmotility leads to delayed treatment and interventions. Prolonged food bolus transit and stasis promote mucosal inflammation and remodeling, subsequently leading to neoplastic changes. We hope to increase awareness among emergency and internal medicine physicians of the prevalence of esophageal dysmotility disorders among patients with rheumatologic disease, and SLE specifically, to improve timing of diagnosis and interventions.

## Introduction

Rheumatologic conditions can present with gastrointestinal (GI) manifestations. A prospective study performed in the US revealed that approximately 9% of patients undergoing high-resolution manometry (HRM) have underlying rheumatologic conditions, with systemic sclerosis being the most common (2.7%), followed by rheumatoid arthritis (2%) and systemic lupus erythematosus (SLE; 1%) [1]. A report by Sultan et al. suggested that the prevalence of esophageal symptoms in patients with SLE varies widely. Chest pain occurs in up to 50%, reflux in 11-50%, and dysphagia in 1-13% of cases [2]. Prevalence of esophageal motility disorders among patients with rheumatologic conditions is an overwhelming 61.1% with absent contractility (AC) present in 28%, ineffective esophageal motility (IEM) present in 21%, and esophagogastric outflow obstruction (EGJOO) present in 12% [1]. As illustrated by this epidemiological data, esophageal dysmotility is very prevalent among patients with rheumatologic disease, but it remains under the radar of emergency or internal medicine clinicians when evaluating patients with dysphagia, chest pain, or heartburn because only a small fraction of patients presenting with these symptoms have an esophageal motility disorder due to underlying rheumatic disease. We hope to bring more awareness to such cases in hopes to avoid delays in diagnosis and treatment. This article was previously presented as a poster at the 2021 ACP Northern Illinois Chapter on October 15th, 2021.

## Case presentation

**Figure 1:**
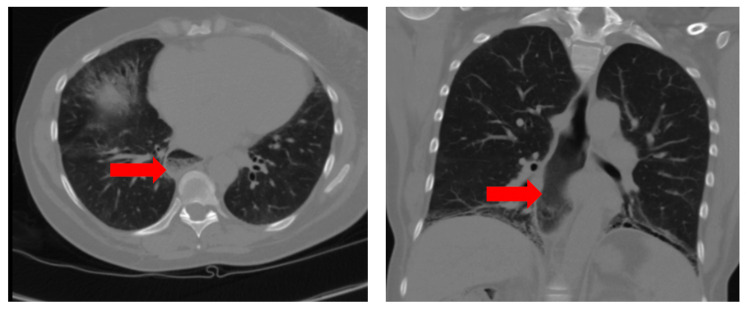
CT сhest demonstrating dilation of the esophagus filled with debris (red arrows) in axial section (left) and coronal section (right)

A 52-year-old female with history of benign hepatic hemangioma, hepatic and splenic hemosiderosis, SLE complicated by lupus nephritis, and pericarditis that required a pericardial window, presented to the outpatient GI clinic with concern of persistent chest pain and difficulty swallowing which were ongoing for at least two years. The pain was previously thoroughly investigated for cardiac etiology, but diagnostic tests resulted negative. She had a hospitalization immediately prior to this presentation for ongoing symptoms of chest pain and dysphagia. During hospitalization, she had a computed tomography angiography (CTA) done as part of pulmonary embolism workup, which was negative for pulmonary or cardiac pathology but revealed a debris-filled appearance of the esophagus with narrowing at the gastroesophageal (GE) junction. Interestingly, this was not the first radiologic evidence of esophageal dilation with CT chest from two years prior (done for pulmonary nodule follow-up) and CT chest from one month prior (done for interstitial lung disease (ILD) workup) also showing dilation of the esophagus (Figure 1).

The CTA done on most recent admission finally prompted further diagnostic evaluation of the GI system. HRM was remarkable for complete absence of esophageal body peristalsis with normal lower esophageal sphincter (LES) resting and slightly reduced relaxation, incomplete upper esophageal sphincter (UES) relaxation, and a small hiatal hernia. Esophagogastroduodenoscopy (EGD) with bougie dilation was performed and was remarkable for erosive gastritis and surgical pathology from the esophagus with unremarkable squamous mucosa with no intraepithelial eosinophils. Patient followed up for pH impedance test, which revealed abnormal distal esophageal acid exposure in upright and recumbent positions, resulting in Demeester score of 245.1. Notably, symptom analysis revealed that six out of seven chest pain episodes were correlated to acid reflux events. Finally, a repeat HRM confirmed more than 50% ineffective esophageal contraction, which was confirmatory for ineffective esophageal motility per Chicago Classification v4.0 [3].

## Discussion

Our patient presented with the unique combination of all three esophageal manifestations of chest pain, dysphagia, and reflux partially responsive to proton pump inhibitors (PPIs). While this combination is rare, it is not specific to SLE-associated esophageal dysmotility, which is why establishing the correct diagnosis took a long time. We specifically wanted to bring attention to the fact that before the CTA on the last admission, there was prior radiographic evidence of esophageal dilation on CT chest done two years prior as part of lung nodule follow-up and CT chest done one month prior for ILD evaluation. Despite patient having radiographic evidence of dilated esophagus filled with debris as early as two years prior, no GI workup was initiated until CTA indicated signs of esophageal disease. We wonder if increasing awareness of emergency and internal medicine clinicians regarding the prevalence of esophageal dysmotility in patients with rheumatic disease, and SLE specifically, may help avoid delays in diagnosis and treatment.

Cardiac causes of presenting symptoms usually take priority and need to be ruled out first, especially as we know that SLE can affect both pericardium and myocardium [4]. However, once they are ruled out, other etiologies need to be given consideration. If patient has a known SLE, given the prevalence of esophageal dysmotility in this patient population, this etiology needs to be investigated. Esophageal manometry abnormalities, particularly hypoperistalsis and aperistalsis, have been observed in up to 72% of patients with SLE [5]. In many instances, functional and structural involvement of the esophagus in patients with rheumatic disorders requires a high index of suspicion for an early diagnosis, correct assessment, intensive surveillance, and aggressive therapy to avoid significant damage to the mucosa and effect on the quality of life. Significant recent advances in the understanding of esophageal pathophysiology, the development of diagnostic techniques, progress in diagnostic and therapeutic endoscopy, and minimally invasive surgery allow early detection and effective long-term therapy for esophageal dysfunction associated with rheumatic diseases [6]. 

Longitudinal findings with radiographic evidence of esophageal debris that are not followed up with may result in exaggerated symptoms such as worsening heartburn, dysphagia, and atypical chest pain, significantly affecting patient’s quality of life. The clinical importance of heartburn in the patient with rheumatic disease relates to underlying gastroesophageal reflux disease (GERD) leading to ulceration, stricture formation, or Barrett's esophagus from long-term exposure [6]. Additionally, continuous saliva stasis and food decomposition in the esophagus may lead to chronic hyperplastic esophagitis. Inflammation may contribute to cancer development through several different ways, including angiogenesis, DNA damage, promotion of cellular growth and multiplication, and dysregulation of programmed cell death [7]. In another study examining esophageal dysmotility, specifically achalasia, Tustumi et al. found that incidence of squamous cell carcinoma was 312.4 cases per 100,000 patient-years at risk. The incidence of adenocarcinoma was 21.23 cases per 100,000 patient-years at risk in patients with achalasia [8]. Although not specifically related to absent contractility, the findings show that long-term esophageal motility abnormalities may be a potentially contributing factor to developing neoplastic changes.

## Conclusions

Esophageal dysmotility leads to prolonged food bolus transit and stasis, which ultimately leads to mucosal changes and predisposes patients to more inflammation and neoplastic changes over time. Increasing awareness of the prevalence of esophageal dysmotility in patients with SLE and other rheumatologic conditions among emergency and internal medicine clinicians could help expedite diagnosis and start treatment earlier, preventing those changes and help improve quality of life by managing symptoms.
